# Publisher Correction: Raymond Gosling: the man who crystallized genes

**DOI:** 10.1186/s13059-023-02946-5

**Published:** 2023-04-25

**Authors:** Naomi Attar

**Affiliations:** Genome Biology, BioMed Central, 236 Gray’s Inn Road, London, WC1X 8HB UK

**Correction: Genome Biol 14, 402 (2013)**

**https://doi.org/10.1186/gb-2013-14-4-402**

Following publication of the original article [1], the author of the interview was informed that Photo 51 shown as Fig. 3 in the article was taken by Raymond Gosling. At that time Raymond Gosling confirmed to Genome Biology that he did physically take the image under the guidance of Rosalind Franklin. This was clarified with a comment added to the online version of article at the time, which has since disappeared. The corrected Fig. 3 legend is published in this correction article.

**Fig. 3 Photo 51.** Raymond Gosling’s Photo 51 of 'B' form DNA taken under the guidance of Rosalind Franklin, which was the highest quality X-ray diffraction pattern of DNA at the time, and an important contribution to Watson and Crick's work on the double helix. © Nature Publishing Group; reproduced with permission.
